# Can the Intestine Perform Some Functions of the Kidney?

**DOI:** 10.1100/tsw.2007.281

**Published:** 2007-11-26

**Authors:** Uday Sankar Chatterjee, Gopal Samanta, Pallab Pradhan, Provat K. Samanta, Tapan K. Mondal

**Affiliations:** ^1^ Park Children Center for Research and Treatment Shree Vishudhanand Hospital and Research Institute, Kolkata, India; ^2^ Zoological Garden, Alipore, Kolkata, India; ^3^ School of Bioscience and Bioengineering, Indian Institute of Technology, Mumbai, India; ^4^ Department of Veterinary Surgery and Radiology, West Bengal University of Animal and Fishery Science, Belgachia, Kolkata, India; ^5^ Department of Veterinary Pharmacology and Toxicology, West Bengal University of Animal and Fishery Science, Belgachia, Kolkata, India

**Keywords:** chronic renal failure, creatinine clearance, end-stage renal disease, intestine, colon, kidney, surface area of gut, dialysis, enteral dialysis, gut dialysis, intestinal dialysis, appendicostomy, antegrade enema, colonic lavage

## Abstract

The majority of patients in countries like India and Pakistan with end-stage renal disease (ESRD) die without renal replacement therapy due to lack of adequate resources. The use of the intestinal mucosa as a semipermeable membrane for removal of urea and creatinine from the body has been previously studied using various types of intestinal lavage for gut dialysis. This study was undertaken in an animal model to assess the applicability, cost of therapy, and acceptability of the method for potential application in humans. Renal failure was induced in six dogs by bilateral ureteric ligation along with six healthy controls. Dialysis fluid was introduced per rectum as an enema, which was repeatedly administered. Clearances of serum creatinine and urea were assessed. Mean recovery of creatinine and urea in dialysate in the present study was around 8.925 mmol/l and around 207.74 μmol//l, respectively. The mean clearances of serum creatinine and urea were, respectively, 0.0683 and 0.0633 ml/sec. Enteral dialysis was effective and, considering its minimal cost (monthly cost will be around US$35–40) vis a vis available methods, it holds promise for the treatment of patients with ESRD. The creation of an appendicostomy for repeated introduction of antegrade enemas would be a consideration.

### INTRODUCTION

The worldwide rise in the number of patients with chronic kidney diseases (CKD) and consequent end-stage renal disease (ESRD) necessitating renal replacement therapy is threatening to reach epidemic proportions over the next decade and only a few countries have robust economies to face the imminent challenges[1]. Kidney transplantation is the best option for ESRD patients, with hemodialysis or peritoneal dialysis being alternatives to transplantation. All these therapies are prohibitively expensive and the majority of the world's population cannot afford them[2]. This is particularly true for the patients in developing and underdeveloped countries, where more than 90% of patients die or are forced to discontinue dialysis treatment within 3 months due to economic reasons[3].

Dialysis involves the transfer of solutes across a semipermeable membrane from blood into a dialysis fluid and has been studied since 1877. Various biological membranes, such as peritoneum, pleura, and gastrointestinal tract have been utilized[4,5]. In 1930, hemodialysis was developed in animals[6] and, in 1959, peritoneal dialysis was re-explored[7].

The total peritoneal surface area (parietal and visceral) is nearly equal to the body surface area and is about 1.78–2.2 $m^2$[8]. Total length of the small intestine is about 280 cm and the mucosal surface area is about 250 $m^2$, approximately equal to a tennis court due to the presence of villi and microvilli[9]. Theoretically, the mucosal surface area of the small gut of 3 cm is equal to that of the entire peritoneum. The length of the large gut from the cecum to the proximal rectum is 100–150 cm. The diameter of the large gut gradually decreases from the cecum (7.5–8.5 cm) to the sigmoid colon (2.5 cm) and it is devoid of villi[10]. In contrast to enterocytes of the small intestine, colonocytes are unable to absorb significant amounts of glucose or amino acids[11]. The colon secretes $K^+$ and $HCO_3^-$ and absorbs $Na^+$, $Cl^-$, and $H_2O$[12], and its capacity is about 1.5–2 l. However, the surface areas of the colon and rectum have not been reported in the available literature.

In 1951, proximal and distal jejunostomies were done with an isolated 2.5-m segment of jejunum in an anephric patient presenting with anuria, with the lavage instilled into that segment. Continuous flow irrigation with dialysate (500–2100 ml/h) was maintained for 10–11 h daily. Urea excretion was 0.0714–0.4641 mol/day. Ultrafiltration or volume removal of around 1300–2100 ml/day was achieved by the addition of sucrose. Serum creatinine level reach steady state with this lavage for 40 days and the patient survived for 46 days[13].

In 1969, a female patient was similarly treated with a better outcome as the urea and creatinine clearances were 0.133–0.2 and 0.108 ml/sec, respectively. It was proposed to avoid this procedure on patients with persistent oliguria and creatinine clearances less than 0.03 ml/sec[14].

Rosin used isolated loops of sheep colon for the same purpose and recovered 2.6 mmol/l of urea from dialysate. Average clearance of urea was 0.045 ml/sec. The author stressed the isolation of the intestinal loops from the gastrointestinal tract to avoid mixing of the dialysate with intestinal content, and for getting better excretion of urea[15].

To date, studies have been done mainly with isolated loops of jejunum or colon. Considering the surface area, loops of small intestine seem to be preferable for this type of dialysis. However, for the creation of a loop of jejunum or colon, operative morbidity and mortality is a limitation, particularly for patients with renal failure, and use of the entire gastrointestinal tract for this purpose may not be feasible for obvious reasons.

In conventional enema (retrograde), solution is instilled into the rectum, but it may not traverse the whole colon upwards; hence, the bowel evacuation is not adequate. To overcome this problem, Malone et al. did an appendicostomy and the lumen of the appendix was used for introduction of a small-sized catheter into the large gut. Through that catheter, tap water was infused slowly as an antegrade enema for relief of constipation in children[16].

It is proposed that enteral dialysis in humans can be performed in the form of a colonic lavage in patients of ESRD with an appendicostomy as the portal for antegrade introduction of the dialysis fluid. The present animal study was conducted on dogs, where a rectal enema was used for introduction of the dialyzing fluid with a view to ascertaining the feasibility, efficacy (solute clearance), untoward effects, and associated costs.

## MATERIALS AND METHODS

### Animals

Twelve apparently healthy adult mongrel dogs of either sex, between the ages of 2–6 years, weighing 11–15 kg, and having an average body surface area of 0.52 $m^2$(range: 0.47–0.62 $m^2$) were considered. All the animals were locally procured and were acclimatized for 3 weeks in a segregated place in the animal house. Clinical evaluation was made before starting the experiment and the animals were considered suitable for the experiment after being found in a healthy condition. The study was undertaken after obtaining prior approval from the Institutional Animal Ethical Committee (IAEC). Twelve dogs were randomly allocated to serve as control (group I) or experimental groups (group II), each group was comprised of six animals.

### Procedures

- Induction of renal failure — Midventral laparotomy was done in animals of both group I (as sham operation) and group II. Temporary reversible uremia was induced in all group II animals by bilateral ligation of the ureters.
- Enteral dialysis — Colonic lavage with peritoneal dialysis fluid (Table 1) was performed in animals of both groups. In group II, enteral dialysis was started 48 h after ureteric ligation, which was retained during the period of dialysis. The total period of enteral dialysis was divided into two broad phases; namely, the first phase for 12 h and the second phase for the next 12 h. In each phase, dialysis fluid was repeatedly instilled four times. The dialyzing fluid (200 ml) was infused into the colon through a Ryle’s tube of 18f and retained for 20 min by pressing the base of the tail against the anus and effluent was retrieved syphonically. This process was repeated five times at a stretch. Thus, a total of 1 l of dialyzing fluid was infused. All the fluid recovered was mixed and 2 ml was used for estimation of creatinine and urea. The same cycle was repeated and a total of 8 l of dialyzing fluid was infused during the period of 24 h.
- Measurement of urea and creatinine of the effluent — Effluent from colonic wash was centrifuged (2000 rpm × 5 min) and supernatant collected for measurements of creatinine and urea, which were done using the autoanalyzer as described by Varley[17].
- Reversal of renal failure — The animals in group II had the ligated ureters relieved by reopening the laparotomy wound 24 h after the colonic lavage. Two dogs developed ureterocutaneous fistula through the laparotomy wound and 11 dogs survived after the procedures. One dog of group II died due to septicemia.

**TABLE 1**
**Composition of Dialysis Fluid**

| | Each 100 ml Contains (g) | In mmol/l |
|---|---|---|
| Dextrose | 2.5 | 138 |
| Sodium chloride | 0.5560 | Chloride 100 |
| Sodium acetate | 0.4760 | Bicarbonate(as acetate) 35 |
| Calcium chloride | 0.0220 | Calcium 1.5 |
| Magnesium chloride | 0.0152 | Magnesium 0.75 |
| Sodium metabisulfide | 0.0150 | Sodium 130 |

### Statistical Analysis

All values were expressed as mean ± SE. Statistical analysis was done using SPSS (Statistical Package for Social Sciences) 10. Statistically significant differences within groups were assessed by paired “t” test, while statistically significant differences between groups were assessed by the Fisher’s “t” test. Statistical significance was defined as $p < 0.05$.

### RESULTS

Experimental bilateral ureteric ligation, as performed in the present study, consistently caused an increase in levels of serum creatinine and urea in all group II animals. Approach to the ureters through a midventral laparotomy for bilateral ligation and its subsequent removal was not problematic. Minimal complications were observed at the site of ligation in animals of group II.

Following ureteric ligation, all the animals in group II became dull and depressed for 3–4 h. Thereafter, the animals showed an improvement in their general condition and after 12 h, started taking food and water. After that, the animals showed no abnormal clinical signs for the first 24–36 h, but their condition started to deteriorate thereafter. At this stage, the animals showed signs of discomfort, were reluctant to move, appeared depressed, and there was drastic reduction in their food and water intake. The animals were markedly dull and depressed, and remained in lateral recumbency between 24 and 48 h postligation. Their eyes were moderately shrunken with congested mucous membranes. Animals in group I appeared clinically normal.

Enteral dialysis was started in both groups at 48 h following the surgical procedures. The physical condition of animals of group II started to improve gradually and at the end of this procedure, the animals showed a marked improvement in their physical condition and general appearances. This could be attributed to extrarenal clearance of waste products, and correction of fluid and electrolyte imbalances. No remarkable changes were noted in animals of group I.

Serum creatinine increased significantly at 24 and 48 h following ureteric ligation in animals of group II as compared to preoperative values ($p < 0.01$). It declined at 12 and 24 h postinitiation of rectal enemas with dialysate. However, values remained significantly higher as compared to preoperative values ($p < 0.01$, Table 2 and Fig. 1). No significant changes were observed in animals in group I.

There were no significant changes in serum urea in animals in group I. However, the serum urea increased significantly in group II at 24 and 48 h postligation as compared to preoperative values ($p < 0.01$). Thereafter, it gradually decreased significantly ($p < 0.01$) at 12 and 24 h after initiation of rectal enema with dialysate. However, values as compared to preoperative ones remained significantly higher ($p < 0.01$, Table 2 and Fig. 2).

Blood glucose and serum $Na^+$ level in dogs of group II showed a gradual and significant decrease ($p < 0.01$) up to 48 h after ureteric ligation and then gradually increased at 12 and 24 h postinitiation of rectal enemas. They remained significantly lower than the preoperative values ($p < 0.01$, Table 2 and and Figs. 3 and 4).

The serum $K^+$ level in dogs of group II showed a gradual, but significant, increase up to 48 h postligation, which corroborates the findings of Singh et al. ($p <0.01$)[18]. Thereafter, it gradually and significantly decreased 24 h following rectal enemas ($p < 0.01$), but remained significantly higher than the preoperative values ($p < 0.01$, Table 2 and Fig. 5).

The concentration of urea and creatinine in the recovered dialysis fluid following rectal enemas with dialysate in group II dogs and rectal enemas with dialysate in group I dogs are presented in Table 3. The values for creatinine and urea in dialysate were significantly higher in group II compared to group I ($p < 0.01$). Mean concentrations of urea and creatinine in dialysate were 8.925 mmol/l and 207.74 µmol/l, respectively (Table 3). About 1594.44 µmol of creatinine and 68.54 mmol of urea were recovered in 7680 ml of effluent dialysis fluid from each dog, over an average of 13.3 h (Table 3). The mean clearances of urea and creatinine were around 0.0683 and 0.0633 ml/sec.

**TABLE 2**
**Serum Creatinine (µmol/l), Urea (mmol/l), Glucose (mmol/l), Potassium and Sodium (mmol/l) Level at Different Times in Dogs following Ureter Ligation and Rectal Enema with Dialysate (Values are Mean with SE of Six Replicates)**

| | Time (h) | | | | |
|---|---|---|---|---|---|
| | | Time Interval following Ureter Ligation | | Time Interval following Rectal Enema with Dialysate | |
| | 0 (a) | 24 (b) | 48 (c) | 12 (d) | 24 (e) |
| Serum creatinine (µmol/l) | | | | | |
| Group I | 72.48 ± 1.76 | 75.14 ± 1.76 | 76.02 ± 1.76 | 71.6 ± 1.76 | 74.25 ± 1.76 |
| Group II | 76.9 ± 1.85 | 243.1** ± 6.1 | 584.32** ± 7.07 | 571.94 ± 3.55 | 558.68* ± 4.42 |
| Serum urea (mmol/l) | | | | | |
| Group I | 3.02 ± 0.07 | 2.97 ± 0.09 | 3.01 ± 0.08 | 3.05 ± 0.09 | 3.02 ± 0.07 |
| Group II | 3.17 ± 0.22 | $22.20^{‡‡} \pm 0.55$ | $26.90^{‡‡} \pm 0.5$ | $21.42^{‡} \pm 0.62$ | $17.14^{‡} \pm 0.53$ |
| Blood glucose (mmol/l) | | | | | |
| Group I | 6.11 ± 0.2 | 6.1 ± 0.33 | 6.38 ± 0.12 | 6.2 ± 0.23 | 5.84 ± 0.18 |
| Group II | 6.57 ± 0.05 | $6.14^{\dagger} \pm 0.06$ | $5.65^{\dagger} \pm 0.06$ | $5.9^{\dagger\dagger} \pm 0.07$ | $6.11^{\dagger\dagger} \pm 0.07$ |
| Serum sodium (mmol/l) | | | | | |
| Group I | 151.75 ± 7.91 | 148.33 ± 10.12 | 143.33 ± 10.83 | 149.16 ± 10.18 | 138.33 ± 9.17 |
| Group II | 149.60 ± 1.04 | $142.36^{\varsigma\varsigma} \pm 1.01$ | $134.88^{\varsigma\varsigma} \pm 1.15$ | 138.18±1.16 | $142.01^{\varsigma} \pm 1.00$ |
| Serum potassium (mmol/l) | | | | | |
| Group I | 5.13 ± 0.10 | 5.08 ± 0.11 | 5.13 ± 0.16 | 5.08 ± 0.14 | 5.18 ± 0.16 |
| Group II | 5.15 ± 0.10 | $5.93^{\Delta\Delta} \pm 0.07$ | $6. 61^{\Delta\Delta} \pm 0.10$ | 6.28 ± 0.08 | $5.96^{\Delta} \pm 0.08$ |

*Note:* $^{**}p < 0.01$ compared to “a”, $^{*}p < 0.05$ compared to “c”, $^{‡‡}p < 0.01$ compared to “a”, $^{‡}p < 0.01$ compared to “c”, $^{\dagger}p < 0.01$ compared to “a”, $^{\dagger\dagger}p < 0.01$ compared to “c”, $^{\varsigma\varsigma}p < 0.01$ compared to “a”, $^{\varsigma}p < 0.01$ compared to “c”, $^{\Delta\Delta}p < 0.01$ compared to “a”, $^{\Delta}p < 0.05$ compared to “c”.

In this study, total volume of dialysate was 8 l/dog over 13.33 h (Table 3). The dwell time for dialysate was 20 min and the mean D/P (dialysate/plasma) ratio[19] of urea and creatinine were around 0.57 and 0.40, respectively.

The body weight of dogs in group I were 12.66 ± 0.60 kg at the beginning of the dialysis and 12.70 ± 0.61 kg at the end of the dialysis. On the other hand, the body weight of dogs of group II at the start and the end of dialysis were 13.33 ± 0.33 and 13.36 ± 0.33 kg, respectively. The body weight of both groups (control and experimental) were not significantly different at the end of dialysis.

### DISCUSSION

It has been observed that a rise in salivary creatinine occurs after total nephrectomy in cattle[20,21]. In group I (control), kidneys were functionally normal, and the excretion of urea and creatinine by colonic lavage was not significant (Table 3, Figs. 1 and 2). This confirms our earlier knowledge that excretion of urea and creatinine from the gut is dependent on the condition of the kidneys.

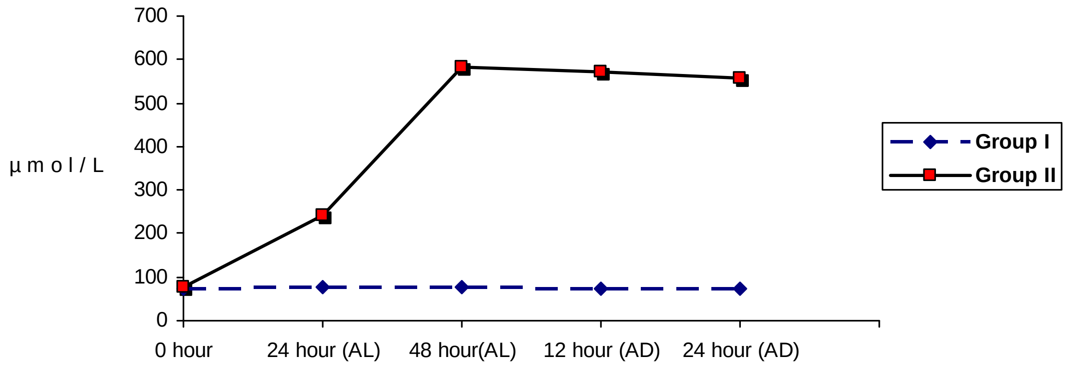

**FIGURE 1**. (SERUM CREATININE): Showing the serum creatinine (µmol / L) level at different time interval. AL = after ligation, AD = after dialysis.

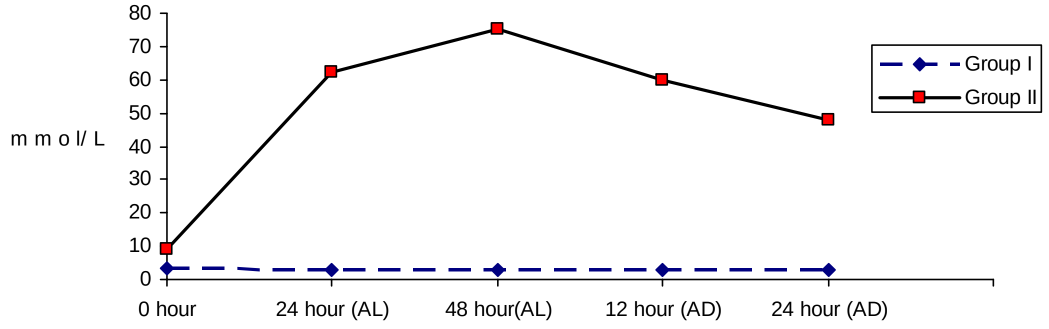

**FIGURE 2.** (SERUM UREA): Showing the serum urea level (mmol/L) at different time interval. AL = after ligation, AD = after dialysis

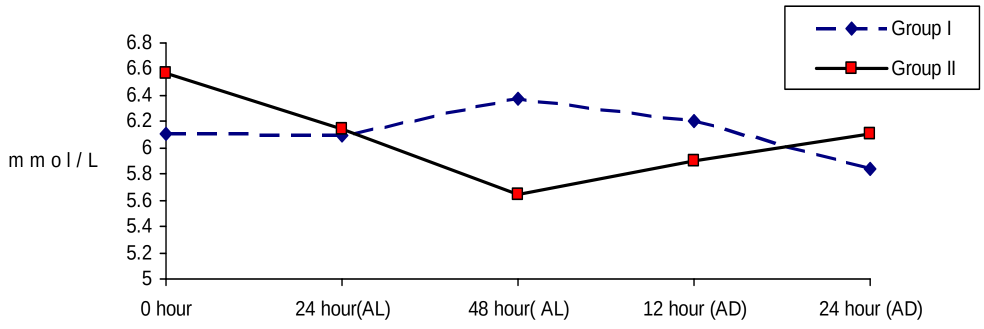

**FIGURE 3.** (BLOOD GLUCOSE): Showing the Blood Glucose level (mmol/L) at different time interval. AL = after ligation, AD = after dialysis.

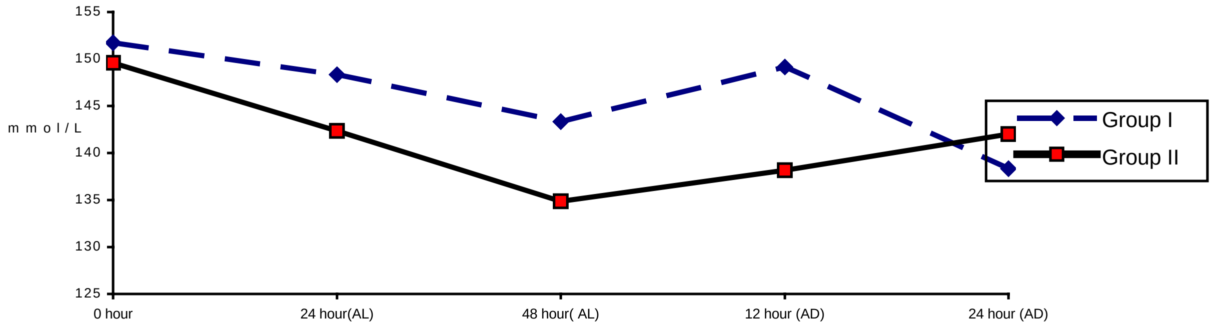

**FIGURE 4.** (SERUM SODIUM): Showing the Blood Glucose level (mmol/L) at different time interval. AL = after ligation, AD = after dialysis.

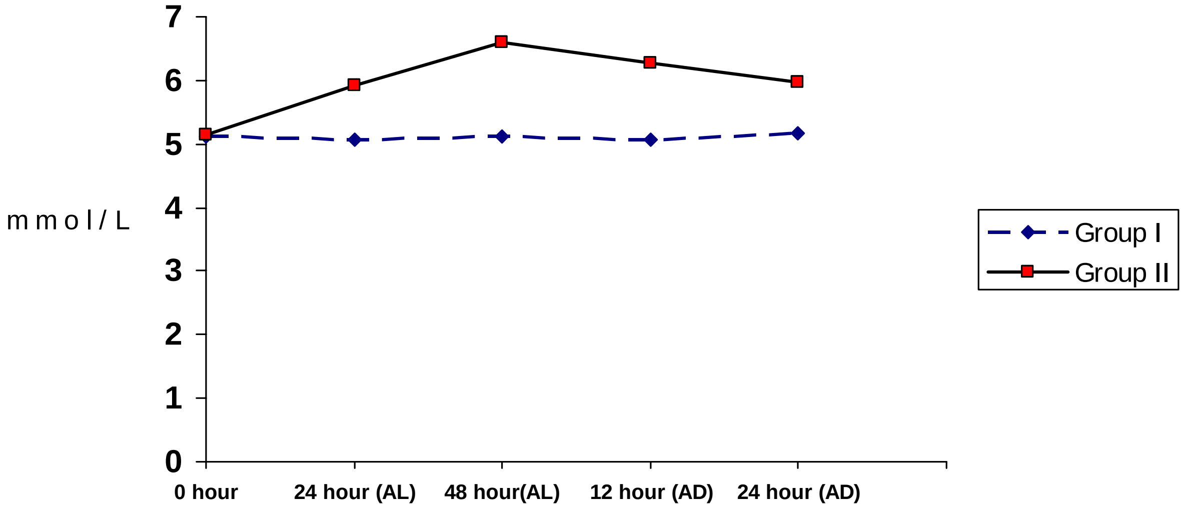

**FIGURE 5.** (SERUM POTASSIUM): Showing the serum potassium (mmol/L) level at different time interval. AL = after ligation, AD = after dialysis

The fall in blood glucose 24 and 48 h after ureteric ligation might be due to continuous anorexia and utilization of reserve stores during starvation. Subsequent rise of blood glucose were due to absorption of glucose from dialysate (Table 2 and Fig. 3).

In contrast to human beings, normal values for serum sodium in dogs range from 143 to 158 mmol/l[22]. Dilutional hyponatremia following ureteric ligation was noted, and following 24 h of enteral dialysis, there was significant ($p < 0.01$) increase in serum sodium level. This could be due to absorption of sodium from dialysate (Table 2 and Fig. 4).

Several studies have shown enhanced secretion of potassium through the rectum and colon in renal failure[23,24,25]. The colonic lavage has enhanced the excretion further and significantly decreased serum potassium (Table 2 and Fig. 5).

Ultrafiltration or volume removal is possible with the system by increasing the concentration and changing the composition of the irrigating fluid[13]. There was a possibility of water retention due to administration of high volume of dialysate; however, retention of water in the animals was not significant in this study. Ultrafiltration was not tried. Solute clearance per week is obviously dependent on the achieved clearances of solute and the total exchange time. In this study, the calculated potential solute clearance would be around 73.6 l/week (Table 4).

**TABLE 3**
**Concentration of Urea (mmol/l) and Creatinine (µmol/l) Levels in Recovered Dialysate Fluid following Rectal Enema with Dialysate in Dogs following Ureter Ligation (Values are Mean with SE of Six Replicates)**

| | First Phase of the Enema (Up to 12 h) | | | | Second Phase of the Enema (12–24 h) | | | |
|---|---|---|---|---|---|---|---|---|
| | First Enema | Second Enema | Third Enema | Fourth Enema | First Enema | Second Enema | Third Enema | Fourth Enema |
| Duration (min) | 100 | 100 | 100 | 100 | 100 | 100 | 100 | 100 |
| Fluid infused (ml) | 1000 | 1000 | 1000 | 1000 | 1000 | 1000 | 1000 | 1000 |
| Fluid recovered (ml) | | | | | | | | |
| Group I | 955.00 ± 9.11 | 931.25 ± 15.49 | 927.50 ± 15.12 | 924.16 ± 13.66 | 945.83 ± 17.17 | 955.83 ± 13.90 | 941.66 ± 15.87 | 956 ± 14.22 |
| Group II | 974.00 ± 21.86 | 951 ± 19.39 | 964 ± 17.45 | 956 ± 13.08 | 948 ± 11.66 | 968 ± 12.29 | 953 ± 9.89 | 966 ± 10.22 |
| Urea (mmol/l) | | | | | | | | |
| Group I | 0.614 ± 0.046 | 0.528 ± 0.046 | 0.564 ± 0.078 | 0.517 ± 0.057 | 0.553 ± 0.067 | 0.610 ± 0.042 | 0.624 ± 0.06 | 0.589 ± 0.057 |
| Group II | 11.12 ± 0.42 | 11.06 ± 0.69 | 11.65 ± 1.14 | 09.75 ± 0.94 | 10.82 ± 1.63 | 7.73 ± 0.47 | 9.40 ± 0.60 | 9.52 ± 0.62 |
| *p* values | <0.001 | <0.001 | <0.001 | <0.001 | <0.001 | <0.001 | <0.001 | <0.001 |
| Creatinine (µmol/l) | | | | | | | | |
| Group I | 13.26 ± 1.41 | 15.02 ± 1.76 | 14.14 ± 1.23 | 13.26 ± 1.06 | 15.02 ± 1.67 | 14.14 ± 1.41 | 15.91 ± 0.88 | 14.14 ± 1.06 |
| Group II | 219.23 ± 20.33 | 249.28 ± 23.86 | 240.44 ± 16.79 | 255.47 ± 15.9 | 234.26 ± 17.68 | 196.24 ± 15.91 | 242.21 ± 14.14 | 241.33 ± 18.56 |
| *p* values | <0.001 | <0.001 | <0.001 | <0.001 | <0.001 | <0.001 | <0.001 | <0.001 |

**TABLE 4**
**Comparative Solute Clearance/Week/1.73m$^2$ (Daily Clearances were Converted to Weekly by Multiplying the Daily Values by Seven)**

| Kidney | Hemodialysis (3 days/week and 4 h/day ) | Peritoneal Dialysis (Daily Dialysis Every 4 h Over 24 h) | In this Study (Dialysis in 13.3 h/day ) |
|---|---|---|---|
| 750 l | 130 l | 70 l | 73.6 l |

Duration of exchange is high in this study. This may be shortened by the application of such techniques as is used in continuous flow peritoneal dialysis (CFPD) as the solute clearance is directly proportional to the volume of exchange[26]. Another factor for effective dialysis is the rapidity of solute transfer from plasma to dialysate and that is measured as D/P ratio. This ratio increases as the dwell time of dialysate increases. In peritoneal dialysis, the mean D/P ratio of urea and creatinine is 0.38 and 0.32 at 0.5 h, and 0.91 and 0.69 at 4 h, respectively. D/P ratio in this colonic dialysis is comparable to peritoneal dialysis. Unlike peritoneal dialysis, long dwell time is not possible in colonic dialysis, but as the cost of dialysate for colonic dialysis is low (explained below); solute clearance may be increased by increasing the frequency as well as the volume of dialysate. It is possible to maintain a continuous inflow through the appendicostomy and a continuous outflow through the anus, similar to CFPD. A similar type of continuous flow intestinal lavage has been studied as mentioned before[13].

Surface area that comes into contact with the dialysis solution in an antegrade enema is more than that in retrograde enema. It has already been mentioned that about 3 cm of small gut has the mucosal surface area equal to the whole peritoneum[9]. In an antegrade enema, there is a possibility that, due to reflux, dialysate will come into contact with ileal mucosa and this may make the enteral dialysis more effective. Antegrade enema by appendicostomy[17] may also be acceptable ergonomically in contrast to repeated retrograde enema. An increase in number, frequency, and volume of exchanges may be more tolerable than the retrograde one.

Maintenance of sterility is of utmost importance in preparation and administration of dialysate in peritoneal dialysis and, hence, the cost of the peritoneal dialysis is higher. In contrast, colonic dialysis fluid can be prepared from potable water and the patient can be trained for self-administration. Estimated monthly cost will be around US$35–40. Further clinical trials for this type of gut dialysis in renal failure, if positive, may be able to extend life where no other means are possible.

### ACKNOWLEDGMENT

We are grateful to Dr. R. Pandey, Associate Professor, Department of Nephrology, I.P.G.MER. and S.S.K.M Hospital, Kolkata, India, for his nephrological consultations and for necessary information made available to us regarding the study.

We are also grateful to Mr. Ashes Mukherjee of Chinsura, Hoogly, West Bengal, India, nephew of one the authors, who was suffering from ESRD and was on hemodialysis, and was waiting for transplantation. He consented (after Institute Ethical Committee Approval) to allow us to perform repeated retrograde enemas of different compositions and concentrations in order to allow us to get an idea about the amount of urea and creatinine that may be extracted. He also participated in the discussion of proposed application of this lavage through appendicostomy.

We are also grateful Dr. (Mrs.) Mitali Chatterjee, Reader, Department of Pharmacology, Calcutta University of Kolkata, for her sincere help in correction and revision of the manuscript.
